# Kinetics of programmed and spontaneous ribosome sliding along the mRNA

**DOI:** 10.1093/nar/gkae396

**Published:** 2024-05-23

**Authors:** Tamara Senyushkina, Ekaterina Samatova, Maria Klimova, Marina V Rodnina

**Affiliations:** Max Planck Institute for Multidisciplinary Sciences, Department of Physical Biochemistry, 37077 Göttingen, Germany; Max Planck Institute for Multidisciplinary Sciences, Department of Physical Biochemistry, 37077 Göttingen, Germany; Max Planck Institute for Multidisciplinary Sciences, Department of Physical Biochemistry, 37077 Göttingen, Germany; Max Planck Institute for Multidisciplinary Sciences, Department of Physical Biochemistry, 37077 Göttingen, Germany

## Abstract

The ribosome can slide along mRNA without establishing codon-anticodon interactions. This movement can be regulated (programmed) by the elements encoded in the mRNA, as observed in bypassing of non-coding gap in *gene 60* of bacteriophage T4, or occur spontaneously, such as during traversal by the 70S ribosome of the 3′UTRs or upon re-initiation on bacterial polycistronic genes. In this study, we investigate the kinetic mechanism underlying the programmed and spontaneous ribosome sliding. We show that the translation rate of *gene 60* mRNA decreases as the ribosome approaches the take-off site, especially when the KKYK regulatory sequence in the nascent peptide reaches the constriction site in the ribosome exit tunnel. However, efficiency of bypassing increases when the ribosome traverses the gap quickly. With the non-coding gap exceeding the natural 50 nt, the processivity of sliding remains high up to 56 nt, but drops sharply beyond that due to the loss of mRNA elements support. Sliding efficiency is temperature-dependent; while temperature regulates the number of ribosomes initiating programmed bypassing, traversing the long gaps becomes increasingly unfavorable at lower temperatures. This data offers novel insights into the kinetic determinants of programmed and spontaneous ribosome sliding along the mRNA.

## Graphical Abstract

**Graphical Abstract:**
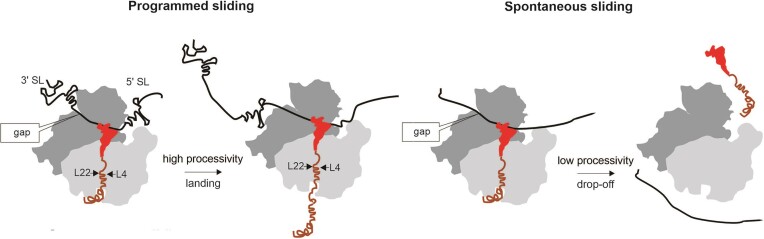

## Introduction

The ribosome is a molecular machine that moves along its mRNA track in steps of three nucleotides (codons) each time a new amino acid is added to a growing peptide. This conventional form of translocation is facilitated by the elongation factor G (EF-G) at the cost of GTP hydrolysis. Alternatively, the ribosome, or its small ribosomal subunit, can move along the mRNA without decoding. In eukaryotes, small ribosomal subunits move along (scan) the mRNA’s 5′ untranslated region (5′UTR) in search of a start codon, which is pivotal for translation initiation. In bacteria, sliding of small ribosomal subunit can aid in translation initiation (1) and in the re-initiation on successive open reading frames (ORFs) within polycistronic transcripts (2,3). Also the 70S ribosomes can slide along the polycistronic mRNA, leading to random initiation events from non-canonical start codons downstream of the termination site of the preceding ORF (4), although the mechanism is likely different from that employed during scanning by the small ribosomal subunit. Eukaryotic 80S ribosomes can slide along the mRNA 3′UTR (5–8) or along large intergenic non-coding RNAs (lincRNAs) (9). One remarkable instance of sliding is the bypassing of a 50-nucleotide non-coding region in *gene 60* of bacteriophage T4 (10). Similar bypassing events are found in several mitochondrial genes of yeast *Magnusiomycetes* (11). The mRNA of *gene 60* has a propensity to form extensive secondary structures (12), some of which are crucial for bypassing (10,13–17). Spontaneous sliding over short distances can occur under conditions of tRNA scarcity (18,19). A translation pause seems to be a common prerequisite for the initiation of ribosome sliding (13,20).

**Figure 1.**
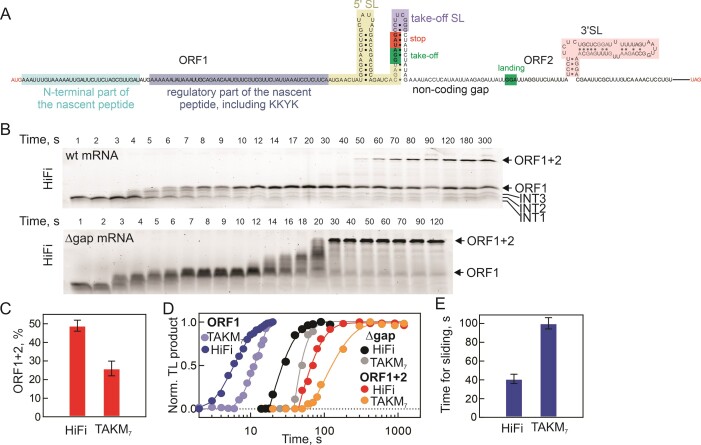
Translation of *gene 60* mRNA *in vitro*. (**A**) The sequence of g*ene 60* mRNA with regulatory elements that promote bypassing. After translating ORF1 (46 aa), the ribosome detaches from the GGA codon (green box) upstream of the proximal stop codon (red box) in the take-off SL (lilac) and bypasses a 50-nt non-coding gap until reaching the landing GGA codon (green box). The major regulatory elements are the sequence of the nascent peptide (shaded gray area), the 5′SL in the mRNA (yellow), the take-off SL and the 3′SL (light red) (13–15). (**B**) Time courses of translation for the full-length and Δgap mRNAs in HiFi. Translation products were separated by SDS-PAGE and detected using the fluorescent reporter (BodipyFL) attached to the N-terminal Met in the peptide. Translation of ORF1 is terminated at the UAG stop (ORF1 product); ORF1 + 2 is the product of ribosome bypassing and translation to the end of the ORF2. Δgap (160 aa) is the construct containing ORF1 + ORF2 without the proximal stop codon and the non-coding gap. (**C**) Bypassing efficiency in HiFi and TAKM_7._ Shown are mean values with error bars representing the range of values from two independent experiments. (**D**) Time courses of translation (TL) in HiFi and TAKM_7_ buffers resulting in ORF1, ORF1 + 2 and Δgap peptides. (**E**) Time of sliding in HiFi and TAKM_7._ Shown are mean values with error bars representing the range of values from two independent experiments.

The mRNA of the bacteriophage T4 encoding *gene 60* contains two open reading frames (ORF1 and ORF2) separated by a 50-nucleotide non-coding gap (10) (Figure 1A). Initially, translation of ORF1 proceeds rapidly but then slows down until it halts at the take-off GGA codon followed by the UAG stop codon (13,15). In this stalled state, the mRNA downstream of the take-off codon in the P site forms a small stem-loop (SL) in the A site (21), while the nascent peptide establishes numerous contacts with the ribosome's peptide exit tunnel (21). The A-site SL and the interactions of the nascent peptide promote the formation of a non-canonical hyper-rotated conformational state of the ribosome (13,22) and lock the uninduced inactive state of the peptidyl transferase center, preventing premature termination or read-through at the take-off site (21). Upon binding of EF-G–GTP, the A-site SL undergoes pseudo-translocation, leading to GTP hydrolysis, unlocking the mRNA and disruption of the codon-anticodon interactions of the peptidyl-tRNA in the P site, thereby enabling the ribosome to initiate sliding along the mRNA (22). The mRNA in the non-coding region remains largely unfolded (12,14). While sliding, the ribosome adopts a rotated conformation, with EF-G continuing to hydrolyze GTP at a rate of 1.8 GTP molecules per nucleotide of the non-coding gap (22). As the ribosome advances along the mRNA, the regions corresponding to the 5′SL and the take-off SL emerge from the ribosome and begin to fold, promoting directional sliding through the non-coding gap. A secondary structure at the beginning of ORF2 (3′SL) facilitates ribosome landing (14). Approximately 50% of ribosomes that initiate bypassing subsequently resume translation of ORF2, primarily due to spontaneous drop-off of the peptidyl-tRNA^Gly^ during sliding (13,14,23). In the absence of codon-anticodon interactions, peptidyl-tRNA remains associated with the ribosome through contacts with the nascent peptide. Following landing, the ribosome remains in a rotated conformation until the next aminoacyl-tRNA, Leu-tRNA^Leu^, enters the A site (22), resulting in an additional delay before translation of ORF2 can begin (13).

While we are beginning to understand the factors triggering ribosome sliding, we still lack detailed molecular insights into this process compared to the well-studied canonical translocation. Initial insights into translation kinetics have been provided by single-molecule FRET (smFRET) studies, which track ribosome transitions between the non-rotated and rotated states (13). However, the relatively slow translation rates in the smFRET experimental setup (e.g. translation of the first 30 codons requiring 200–250 s, rather than the expected ∼10 s) may mask some of the granularity in the translation/bypassing process, which leaves open a number of questions. When do rapidly translating ribosomes switch to pausing and is there a correlation between rapid/slow translation and efficient bypassing? How fast is the sliding process itself? How processive are ribosomes during sliding? What is the average distance over which a ribosome can slide? How does mRNA structure influence sliding? Is there a mechanistic difference between programmed and spontaneous sliding? Here, we aim to address these questions through kinetic analysis of bypassing using the reconstituted *in vitro* translation system from *Escherichia coli*. The results of this study offer insights not only into the process of programmed ribosome bypassing but also shed light on the fundamental mechanisms underlying spontaneous ribosome sliding along mRNA.

## Materials and methods

### Buffer and reagents

All biochemical experiments were carried out in HiFi buffer (50 mM Tris–HCl, pH 7.5, 70 mM NH_4_Cl, 30 mM KCl and 3.5 mM MgCl_2_, 8 mM putrescine and 0.5 mM spermidine) or TAKM_7_ (50 mM Tris–HCl, pH 7.5, 70 mM NH_4_Cl, 30 mM KCl and 7 mM MgCl_2_) (14,24). Total *Escherichia coli* tRNA was from Roche. Site-directed mutagenesis of the *gene 60* construct (15) was performed using the Quick-change PCR protocol (14). mRNAs were produced by T7 RNA-polymerase *in vitro* transcription and purified by ion-exchange chromatography on a HiTrapQ HP5 ml column (GE Healthcare). 70S ribosomes and other translational components prepared from *E. coli* according to the published protocol (25–27). Fluorescence labeling of ribosomal subunits is described in detail (28). The extent of subunit labeling was determined spectroscopically and was close to 100%, the activity was tested as reported (28,29).

### *In vitro* translation

**Figure 2.**
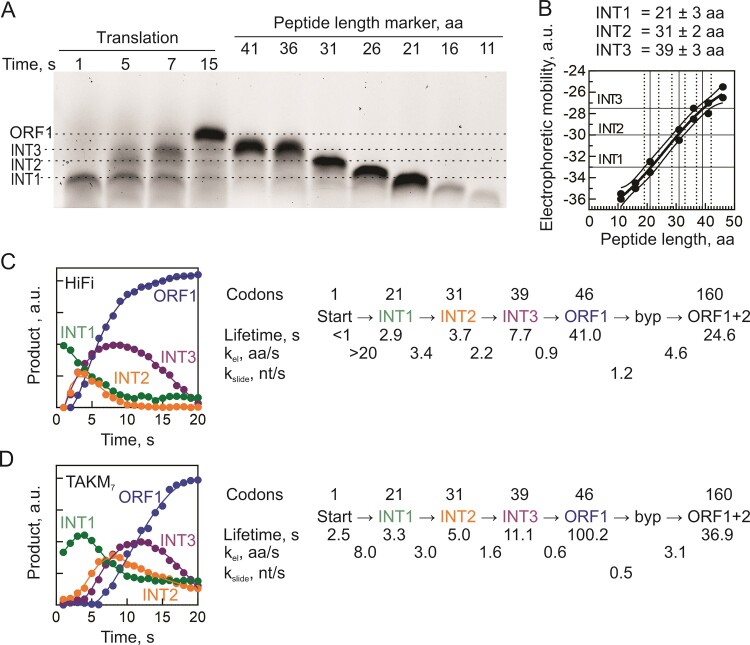
Gradual translation slowdown upon ORF1 translation. (**A**) Identification of the peptide length of translation intermediates (INT1, INT2 and INT3) by comparison of the SDS-PAGE mobility of INT1-INT3 with the peptide markers prepared by run-off translation of *gene 60* ORF1 of indicated length. (**B**) Quantification accuracy for the peptide length of pausing intermediates. Symbols represent the upper and lower border of peptide in the left panel, bold line is the average used for the estimation of the INT length. (**C**) Time courses of translation of INT1 to INT3 and ORF1 in HiFi. Smooth lines represent global fits that yield kinetic parameters shown in the translation model to the right. (**D**) Same as C for translation in TAKM_7_.

Translation was carried out as described (14) with the following modifications. Initiation complexes were formed by incubating ribosomes (0.5 μM), mRNA (1.5 μM), IF1, IF2 and IF3 (0.75 μM each), GTP (1 mM) and either Bodipy-[^3^H]Met-tRNA^fMet^ or initiator f[^3^H]Met-tRNA^fMet^ (0.75 μM) in HiFi buffer for 30 min at 37°C. The ternary complex EF-Tu–GTP–aminoacyl-tRNA was prepared by incubating EF-Tu (58 μM) with GTP (1 mM), phosphoenol pyruvate (3 mM) and pyruvate kinase (0.1 mg/ml) for 15 min at 37°C, then adding purified total aa-tRNA (120 μM) and EF-G (1 μM) and incubating for 1 min at 37°C. *In vitro* translation was started by mixing initiation ribosome complexes (0.08 μM) with the ternary complexes (100 μM) and incubated at temperature range from 10°C to 37°C for 20 min. Products were separated by Tris-Tricine gel electrophoresis (30). Fluorescent peptides were detected after gel electrophoresis using Starion IR/FLA-9000 scanner (FujiFilm) and quantified using the Multi Gauge software. Bypassing efficiency was calculated as a ratio of the density corresponding to the ORF1 + 2 band to the sum of the ORF1 and ORF1 + 2 bands. The identity of the products was confirmed as previously described (14,21). The global fit shown in Figure 2 was performed in Matlab (MathWorks) using the Optimization Toolbox.

### Single molecule experiments using TIRF microscopy

Single molecule FRET experiments were carried out at 22°C. The stalled ribosome complexes were diluted in HiFi buffer to a final concentration of 1 nM and immobilized on biotin-PEG quartz slides pre-incubated with NeutrAavidin (Thermo Scientific) using the mRNA annealed to a biotinylated primer. The imaging buffer was HiFi buffer supplemented with an oxygen-scavenging system (5 mM protocatechuic acid and 50 nM protocatechuate-3,4-dioxygenase from *Pseudomonas*) and a triplet-state quencher mixture (1 mM Trolox and 1 mM methylviologen) (Sigma – Aldrich) as described (31). smFRET experiments were performed on an IX 81 inverted objective-based TIRF microscope with a 100 × 1.45 numerical aperture oil immersion objective (PLAPON, Olympus). A CCD-C9100-13 camera (Hamamatsu) was used for recording images at a time resolution of 10 frames/s. Fluorescence time traces for donor (Cy3) and acceptor (Cy5) were extracted and analyzed using custom-made Matlab (MathWorks) software according to published protocols (31).

## Results

### Kinetics of translation and bypassing

We studied translation kinetics and bypassing efficiency of *gene 60* using the fully reconstituted translation system from *E. coli* (14). To synchronize translating ribosomes, we assembled initiation complexes comprising 70S ribosomes, *gene 60* mRNA, and fluorescence-labeled initiator BodipyFL-Met-tRNA^fMet^ and started translation by adding an excess of ternary complexes EF-Tu–GTP–aminoacyl-tRNA and EF-G–GTP (Methods). After stopping the reaction, translation products were separated by SDS-PAGE and visualized using BodipyFL attached to the N-terminus of the nascent peptide (Figure 1B, Supplementary Figure S1). As the kinetics of translation and bypassing efficiency vary depending on the reaction buffer (14), we first sought to establish the correlation between speed and bypassing efficiency by comparing translation products obtained under two different buffer conditions: the HiFi buffer containing low Mg^2+^ concentration (3.5 mM) together with polyamines spermidine and putrescine (22,24), and the TAKM_7_ buffer containing a higher Mg^2+^ concentration (7 mM) without polyamines (14). Translation of ORF1 yields a short product (46 amino acids (aa)) terminated at the proximal stop codon, while translation of ORF1 + ORF2 produces the bypassing product (160 aa; Figure 1B). Peptide bands below the ORF1 band and between the ORF1 and ORF1 + 2 bands correspond to translation intermediates accumulating due to transient ribosome pausing at discrete locations on the mRNA (Figure 1B, Supplementary Figure S1). The bypassing efficiency is higher in HiFi (49%) than in TAKM_7_ (26%) (Figure 1C). Translation of ORF1 is completed within 10–15 s in either buffer, but occurs faster in HiFi compared to TAKM_7_ (Figure 1D; Supplementary Table S1). The synthesis time of ORF1 + 2 on the wild-type (wt) *gene 60* mRNA is 76 s and 153 s in HiFi and TAKM_7_, respectively. The translation time courses of the construct containing ORF1 + ORF2 without the non-coding gap (Δgap) are about 32 and 53 s, respectively (Figure 1D; Supplementary Table S1). This suggests that translation of both ORFs is somewhat slower in TAKM_7_ than in HiFi, with the translation time for ORF2 of 25 and 37 s, respectively. The difference in translation times between the wt bypassing and Δgap mRNAs reveals how much time the ribosome spends while pausing at the take-off site, sliding, and landing before the translation of ORF2 can resume, about 40 s in HiFi and 100 s in TAKM_7_ (Figure 1E). The shorter bypassing time in the HiFi buffer correlates with the higher bypassing efficiency.

For a more direct comparison with existing smFRET data, we also estimated the time for bypassing using the methodology developed by (13). Specifically, we followed step-wise translation by monitoring ribosome transitions from the non-rotated (decoding) to rotated (translocation) steps using smFRET labels attached to ribosomal proteins S6 and L9 (28,32). As expected, bypassing resulted in a long-lived rotated state (Supplementary Figure S2). The estimated lifetime of bypassing was about 30 s, close to the value estimated from our ensemble experiments, but much shorter than reported in smFRET experiments, 88 s (13). To investigate whether the differences in the bypassing time could be attributed to limiting concentration of EF-G, which is known to trigger bypassing (22), we conducted measurements at various EF-G concentrations. However, we found that the reaction reached saturated even at the lowest concentration we could use without compromising translation. This outcome suggests that the slower bypassing observed in the studies conducted by Chen *et al.* (13) might be indicative of a lower intrinsic activity of the ribosome or different buffer composition in their experimental setup. Such a possibility should be considered when evaluating the rate-limiting steps in the reaction, in particular when studying the kinetics of bypassing or the effects of mutations.

### Pauses during translation of ORF1

While an average elongation rate provides a straightforward measure for ribosome movement along mRNA, the actual progression is more complex, often characterized by periods of rapid translation interspersed with pauses. For ORF1 of *gene 60*, smFRET studies have suggested that ribosomes begin to stall at codons 40 to 45, i.e. approximately 4 codons before the take-off codon (13). In our experimental setup, ribosome pausing leads to transient accumulation of peptides shorter than the ORF1 product (Figure 1B; Supplementary Figure S1). The accumulation of peptide products indicates a gradual slowdown, suggesting that ribosomes start to pause much earlier than 4 codons before the take-off, prompting us to analyze the local elongation rates along the ORF1 mRNA. To pinpoint the sites of pausing, we determined the amino acid length of the corresponding translation products using *gene 60* peptide standards of varying lengths from 11 to 41 amino acids, prepared by *in vitro* translation of truncated mRNAs (Figure 2A, B). The length of the first accumulating intermediate (INT1) is 21 ± 3 amino acids. At this length, the initial part of the nascent peptide signal (K_14_KYKLQNN_21_VRRSIKSSS) has already been translated, and the KKYK motif is expected to reside at the narrowest part of the polypeptide exit tunnel, known as the constriction site. The subsequent pausing site (INT2) occurs after 31 ± 2 amino acids have been incorporated, coinciding with the arrival of the 5′SL at the mRNA entry channel of the ribosome. The final intermediate, INT3, comprises 36–41 amino acids, largely consistent with the observed translation slowdown in smFRET experiments (13). At this length, most of the regulatory portion of the nascent peptide occupies the tunnel, and the 5′SL must unfold to enter the mRNA channel, while the take-off SL likely resides just outside the ribosome. Notably, the positions of pause sites are consistent between the HiFi and TAKM_7_ buffers (Figure 1B and Supplementary Figure S1).

To estimate translation rates across different segments of ORF1, we constructed a kinetic model of translation for global fitting analysis. The best-fitting minimal model comprises five consecutive irreversible steps, corresponding to the formation of INT1, INT2, and INT3, ORF1 and ORF1 + 2 products (Figure 2C,D). In the HiFi buffer, translation starts rapidly but slows down significantly at codon 21 (INT1). Further slowdown occurs at codon 31 (INT2) and again at codon 39 (INT3) as ribosomes approach the take-off site. After take-off, sliding, and landing, the ribosomes remaining on the mRNA track continue translation at an average rate of 4.6 aa/s until the full-length bypassing product is synthesized. A similar deceleration pattern is observed in the TAKM_7_ buffer. Moreover, although translation inherently proceeds faster in HiFi compared to TAKM_7_, ribosomes slow down to nearly the same rate in both buffers after INT1. This indicates that translation on ribosomes nearing the take-off site becomes independent of the decoding and translocation steps (which are Mg^2+^ and polyamine-dependent) and instead becomes constrained by regulatory elements within the bypassing complexes, such as the nascent peptide and the SL elements in the mRNA.

### Kinetic contribution of the regulatory elements in the mRNA

**Figure 3.**
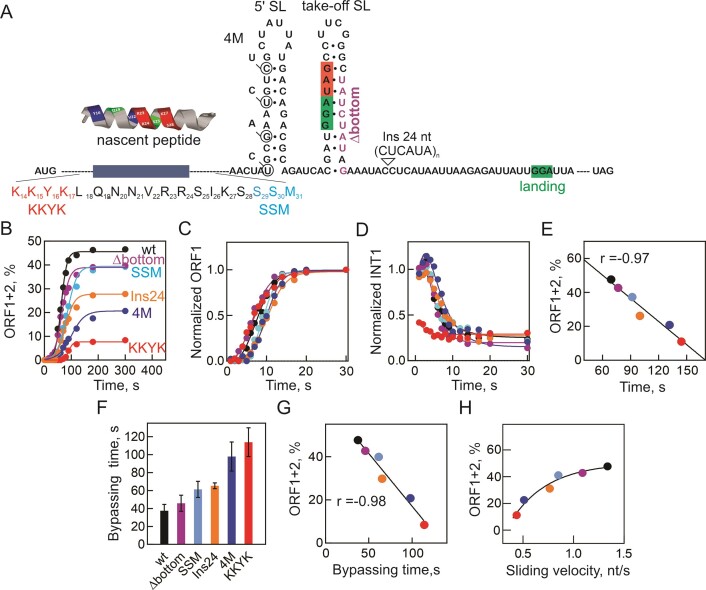
Contribution of the regulatory elements in *gene 60* mRNA to kinetics of ORF1 and ORF1 + 2 translation. (**A**) Mutations in the nascent peptide (KKYK and SSM to AAAA and AAA, respectively); in the mRNA SL elements (4M, which removes the 5′SL without changing the amino acid sequence of the nascent peptide (14)); Δbottom, which removes base pairing in the lower part of the take-off hairpin (13)); and insertions in the non-coding region (Ins 24 (14)). (**B**) Time courses of ORF1 + 2 translation showing the effect of mutations on bypassing efficiency. (**C**) Time courses of ORF1 translation. (**D**) Time courses of INT1 accumulation. (**E**) Correlation between the translation time of ORF1 + 2 and bypassing efficiency. (**F**) Comparison of the bypassing times for different mutants. (**G**) Negative correlation between the duration and efficiency of bypassing. (**H**) Ribosome sliding velocity. The exponential dependence of bypassing efficiency on sliding velocity is marked by a very high Spearman's rank correlation coefficient of ρ = 1, highlighting the statistical significance of the correlation.

Given the pivotal role of mRNA and nascent peptide elements in facilitating bypassing, we next aimed to determine whether these elements also impact translation kinetics. To assess the effect of the nascent peptide, we utilized mRNA mutants that modify its sequence (14). For instance, we replaced the K_14_K_15_Y_16_K_17_ motif with AAAA (the mutant is denoted as KKYK) or the S_29_S_30_M_31_ motif with AAA (denoted as SSM) (Figure 3A). To disrupt mRNA structure, we employed mutants with four synonymous mutations in the 5′SL (4M from (14)) or with an unfolded lower part of the take-off site SL (Δbottom from (13)). Additionally, we utilized mutant mRNAs with an insertion of 24 nt (Ins24) in the non-coding gap (14). This combination of mutations enabled us to cover almost the entire dynamic range of bypassing efficiencies from <10% with the KKYK mutant to 49% with the wt *gene 60* mRNA (Figure 3B) (13–15).

While the overall effect of mutations on the kinetics of ORF1 translation is relatively modest, we observe notable changes in the accumulation of intermediates with the KKYK mutant (Figure 3C, D; Supplementary Figure S3). The mutation of the KKYK sequence reduces stalling at INT1 but results in the accumulation of multiple intermediates of varying lengths between INT1 and INT3 (Figure 3D, Supplementary Figure S3). These additional transient pausing events may be caused by the interactions of the exit tunnel with the nascent chain residues downstream of KKYK, for example with the SSM motif (21). However, the SSM mutation does not affect INT1 accumulation and has minimal kinetic effects (Supplementary Figure S3). This suggests that the KKYK interactions elicit ribosome pausing at INT1, whereas the contribution of the downstream residues in the nascent chain becomes visible only when KKYK is absent. Mutations disrupting putative SL mRNA structures or changing the gap length have limited impact on INT accumulation (Supplementary Figure S3). These findings are consistent with the idea that the accumulation of INT1 and INT2 is influenced by the interactions of KKYK with the exit tunnel, while mRNA secondary structures appear to have minimal effect on the observed translation slowdown (Figure 3). While the effects of mutations on ORF1 translation rates are moderate, the translation time of ORF1 + 2 is significantly altered, correlating with the bypassing efficiency of the respective construct (Figure 3E).

We then estimated the time required for bypassing (Figure 3F), encompassing the total duration for take-off, sliding, and landing. This estimation was based on the translation time courses with different mRNAs, along with the respective times for ORF1 translation and ORF2 translation obtained for the Δgap construct, as described previously for the wt mRNA (Figure 1). On the wt mRNA, ribosomes take approximately 40 s to slide over the non-coding gap, with an average rate of 1.2 nt/s. Mutants impacting bypassing efficiency also extend the time needed for bypassing and reduce sliding velocity. Bypassing efficiency demonstrates a linear correlation with the time required for bypassing (Figure 3G) and an exponential correlation with sliding velocity (Figure 3H). It is likely that when ribosomes slide rapidly, their likelihood of dissociating from the mRNA track is reduced, leading to a larger proportion of ribosomes continuing into ORF2. Given that disruption of any bypassing signals leads to slower and less efficient bypassing, we conclude that the nascent peptide, in conjunction with complex mRNA elements, collectively fine-tunes sliding to achieve high speed, thereby preserving high bypassing efficiency.

### Ribosome's processivity during sliding

**Figure 4.**
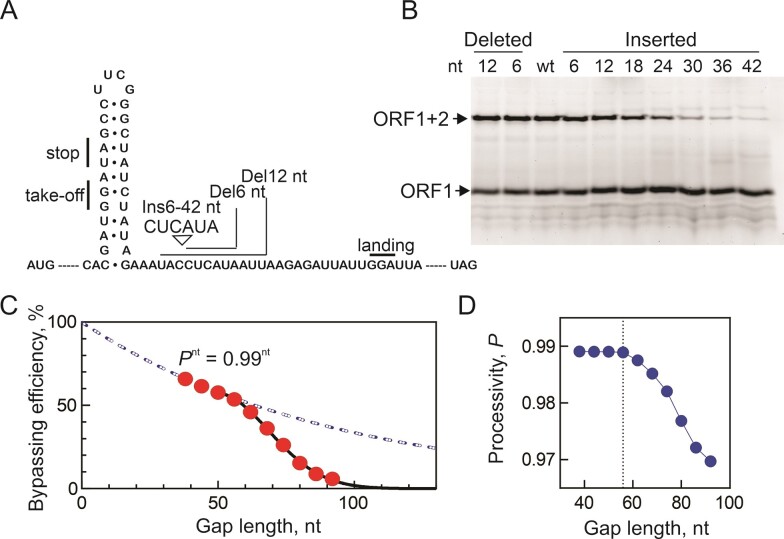
Programmed and spontaneous ribosome bypassing. (**A**) Insertions (Ins) and deletions (Del) in the non-coding gap of *gene 60* mRNA. (**B**) Bypassing efficiency at various gap lengths. (**C**) Two bypassing regimes. At gap length <56 nt, the processivity coefficient *P* obtained by exponential fitting is 0.99, meaning that at each nucleotide, 1% of ribosomes drop-off or stall on the mRNA track, while 99% ribosomes continue sliding. At gap length >56 nt, processivity decreases with each nucleotide passed. (**D**) Processivity coefficients for each gap length. At the lengths <56 nt (dotted line), the processivity is constant. Beyond >56 nt, processivity decreases progressively with gap length.

For effective ribosome bypassing, the accurate placement of regulatory elements relative to the mRNA’s take-off and landing sites is crucial. This precise positioning is a hallmark of programmed sliding. During bypassing, peptidyl-tRNA remains anchored to the ribosome via interactions with the exit tunnel, while the positions of regulatory mRNA elements change and the mRNA structures start to re-fold as the ribosome progresses along the mRNA. In contrast, spontaneous non-programmed bypassing likely occurs without the help of such mRNA elements. To elucidate potential differences between programmed and spontaneous ribosome sliding, we assessed ribosome bypassing efficiency across different mRNA gap lengths ranging from 38 to 92 nucleotides. These variations were achieved by either deleting or inserting up to seven repeats of a 6-nt CUCAUA segment (Figure 4A). Consistent with previous findings (14), we observed a decrease in bypassing efficiency as the gap length increased (Figure 4B, C). Such decrease can result from the ribosome drop-off from the mRNA, moving backwards or stalling on the track; however, given that the ribosome-mRNA complexes are generally unstable in the absence of codon-anticodon interactions, we consider the possibility of prolonged stalling less likely than a ribosome drop-off. Upon closer examination, we identified two distinct kinetic regimes with a breakpoint at approximately 56 nucleotides (Figure 4C). This suggests that ribosomes employ different mechanisms when navigating gaps of shorter versus longer lengths.

To analyze the distance dependence of ribosome sliding, we used exponential fitting. The efficiency of bypassing was estimated as a product of the probabilities of take-off and landing, combined with the efficiency of traversing the non-coding gap. This is expressed by the equation:


\begin{equation*}{\mathrm{Bypassing\ efficiency\ }} = A \times {{P}^{nt}},\end{equation*}

where *A* is the combined probability of take-off and landing and *P* is the processivity coefficient indicating the likelihood of a ribosome moving by one nucleotide (14). Exponential fitting suggested that *A* is close to 100% (Figure 4C, dashed blue line), implying that the majority of ribosomes successfully take off, and those reaching the landing codon continue translation in ORF2, in line with previous reports (14). For gap lengths of 56 nucleotides or less, processivity coefficient *P* was calculated to be 0.99 (Figure 4C, dashed blue line, and 4D), indicating a very high processivity of sliding. Considering that the sliding rate is known from kinetic experiments, and *P* is dependent on the rate constants of forward and backward sliding as well as ribosome drop-off from the mRNA, defined as $P = \frac{{{{k}_{forward}}}}{{( {{{k}_{forward}} + {{k}_{drop - off}} + {{k}_{backward}}} )}}$, we were able to estimate the rates for gap lengths of 56 nucleotides or less : *k_forward_* = 1.2 nt/s and the *k_drop-off_*+*k_back_* = 0.01 nt/s.

For longer gaps (≥56 nucleotides), the processivity decreased progressively (Figure 4D). This decrease could be due to either an increased rate of ribosome drop-off from the mRNA or a higher probability of backward sliding, with the rate constants rising from a combined value of 0.01 to 0.04 nt/s. These changes can be attributed to the growing distance of the ribosome from regulatory mRNA elements, as the interactions with the nascent chain do not change.

### Temperature dependence of ribosome sliding

**Figure 5.**
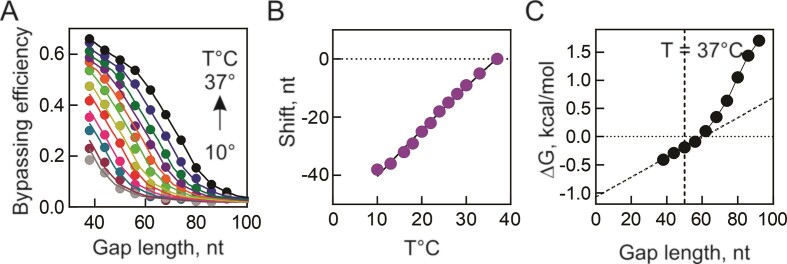
Temperature and gap length dependence of programmed and spontaneous ribosome sliding. (**A**) Bypassing dependence on the gap length measured by different temperatures (from top to bottom, 37, 33, 30, 28, 26, 24, 22, 20, 18, 16, 13, 10°C). Solid lines represent fits to the recurrent formula: byp(*T*,nt) = *P_37_*(nt − *Δ*nt(*T*))^nt-^*^Δnt^****^(T)^***, where *P_37_* is the processivity at *T* = 37°C and Δnt(*T*) is the temperature-dependent shift (in nt) of the effective bypassing distance. (**B**) Temperature dependence of the shift Δnt(*T*). (**C**) Thermodynamic preference for bypassing at 37°C. The Δ*G*° values below 0 (below the dotted line) indicate that bypassing is favorable. Dashed line indicates the gap length in *gene 60* mRNA.

Bypassing can be activated or deactivated by altering the temperature (21), owing to a conformational change at the ribosome's decoding center (22). At low temperature (4°C), the ribosome is stalled in the state before take-off, with peptidyl-tRNA^Gly^ bound to the Gly codon in the P site and the mRNA forming a short helix in the A site (21). To investigate how temperature influences sliding, we conducted experiments measuring bypassing efficiency on mRNAs with increasing gap lengths across temperatures ranging from 37°C to 10°C (Figure 5A). At high temperature, all ribosomes initiate take-off and those that reach the landing site, initiated translation of ORF2 (Figure 4; (14)). The curve has two characteristic phases, one showing high processivity (*P*= 0.99) at gap lengths <56 nt and another with the *P* value decreasing with each additional nucleotide >56 nt in the non-coding gap. As temperature decreases, the maximum bypassing efficiency decreases and the curve shifts towards shorter gap lengths, indicating that fewer ribosomes tend to slide over shorter distances. At lower temperature, very few ribosomes initiate take-off, as indicated by the cryo-EM structure (21) and there is essentially no high-processivity phase (Figure 5, black symbols, *T* = 37°C, points < 56 nt gap). To analyze all data together, we utilized a recurrent formula that estimates the temperature-dependent shift of the bypassing efficiency curve in nt (Figure 5A). The temperature dependence of the shift (Figure 5B) suggests that the length of the traversed gap decreases by 1.5 nt/°C. Notably, because the length-dependence curves do not show the high-processivity component at lower temperature, the calculated temperature shift pertains largely to the spontaneous sliding phase and can be considered an empirical value to predict the distances along which the ribosomes can slide, e.g. for a given fraction of ribosomes that can slide over 80 nt at 37°C, we can predict that at 10°C they will slide only over 40 nt. To elucidate the energetic prerequisites of bypassing (byp), we reanalyzed the data from Figure 5A, converting the values into Δ*G*° utilizing the equation Δ*G*° = −kT*ln(byp/(1 −byp)), where Δ*G*° = 0 signifies 50% bypassing efficiency, and negative values indicate favorable bypassing conditions. In the case of 37°C, the gap length dependence delineates two distinct regimes (Figure 5C). The reaction occurring at 40–60 nucleotides gap length signifies programmed bypassing, a finely-tuned mechanism evolved to maintain a specific level of bypassing efficiency. Conversely, the unfavorable reaction likely characterizes unprogrammed, spontaneous ribosome sliding along mRNA unaided by regulatory mRNA elements.

## Discussion

Ribosome sliding along mRNA remains relatively understudied compared to other forms of translational recoding. An example of programmed bypassing on *gene 60* of bacteriophage T4 demonstrates that different signals in the nascent peptide and the mRNA contribute to the efficiency of bypassing (10,13–15,17,23,33). The present data offer a more detailed understanding of programmed bypassing mechanisms and provide initial insights into the mechanistic aspects of spontaneous ribosome sliding.

During the bypassing of the 50 nt gap in *gene 60* mRNA, ribosomes translate ORF1 up to codon 46. Our analysis of ribosome processivity during sliding indicates that all ribosomes initiate take-off at codon Gly46 and start sliding over the gap and those ribosomes that successfully reach the landing Gly codon continue translation of ORF2 (Figure 4 and (14)). However, not all ribosomes remain on track during sliding due to the spontaneous drop-off or stalling or backtracking events. The average sliding time along the non-coding gap is approximately 30–40 s in HiFi buffer in smFRET and ensemble experiments (Figure 1 and Supplementary Figure S2). Previous smFRET data suggested that during ORF1 translation, ribosomes start to move more slowly from codon 40 to 45 (13). This slowdown was attributed to interactions of the regulatory KKYK motif with the ribosome exit tunnel, suggested to play a pivotal role in initiating take-off. The precise role of the translational slowdown at the onset of bypassing remains unclear, but it aligns with established models of other forms of recoding, particularly frameshifting, where translation slowdown plays a pivotal role in initiating the slippage. However, our experiments, which are performed at the conditions of near-physiological translation rates, provide a more differentiated picture of how speed affects ribosome sliding along the mRNA.

**Figure 6.**
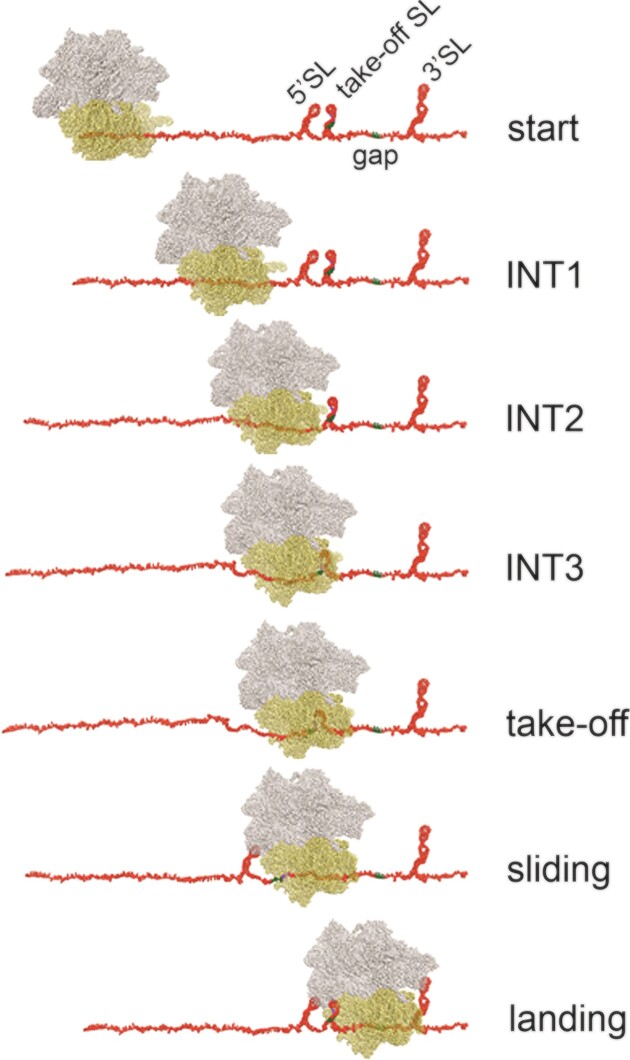
Schematic of ribosome movement along ORF1, take-off and landing. The mRNA is shown without the 5′ or 3′UTR and only the functionally relevant SL structures are indicated (see Figure 1). For further explanations, see text.

We demonstrate that translational slowdown commences early during translation of ORF1, occurring when the nascent peptide reaches a length of approximately 21 aa (Figure 6). At this stage, the KKYK motif likely reaches the ribosome constriction site, which is lined up with positively charged arginine residues (34). Electrostatic repulsion induced by positive charges may contribute to the slowdown via a long-distance effect on the peptidyl transferase center of the ribosome (34). Indeed, mutating the KKYK motif abolishes pausing at INT1, consistent with the notion that KKYK is important for the early stalling, whereas other residues in the nascent chain may attenuate stalling and play a role in anchoring the full-length nascent chain to the ribosome exit tunnel (14,21). This early slowdown of translation may be also important to maintain sufficient spacing between successive translating ribosomes in a polysome to allow the 5′SL to properly fold behind the lead ribosome. In a polysome context, impairments in 5′SL folding can reduce the efficiency of bypassing, resulting in the accumulation of the ORF1 peptide (33).

After passing the first pause site, the ribosomes slow down further when the nascent peptide reaches approximately 31 aa (INT2) and 39 aa (INT3) (Figure 6), corresponding roughly to the arrival of mRNA secondary structure elements, the 5′SL and take-off SL, respectively, at the mRNA helicase center of the ribosome. The partial unfolding of the take-off SL (e.g. by Δbottom mutation) leads to a slightly faster synthesis of the ORF1 product, similarly to the effect seen with the KKYK mutation (Figure 3). However, unlike the KKYK mutation, the Δbottom mutation does not influence the INT1 pause; instead, it reduces the time the ribosome spends at the INT2 site. Disrupting the secondary structure of the 5′SL by 4M mutations does not increase the translation speed, probably because unwinding of these secondary structures is not rate-limiting, as the interactions of the nascent peptide with the tunnel also contribute to the regulation of translation speed.

While the effects of mutations on the average rate of ORF1 translation is small, the estimated sliding time varies depending on buffer conditions (Figure 1) and the presence of mutations (Figure 3). Surprisingly, and in contrast to the expected importance of translational slowdown, we find a linear correlation between higher sliding velocity and bypassing efficiency. In a simplest model, ribosomes that do not slide quickly enough may disengage from the track, resulting in fewer ribosomes reaching the landing site. Depending on the mutant, this effect may be due to a higher drop-off rate, which is likely for nascent peptide mutants including KKYK, or to the relative changes in forward and backward sliding, which may be modulated by secondary structures forming behind the sliding ribosome (14). In this model, translational slowdown before the take-off would not directly regulate sliding, but rather represents side effects of the regulatory elements, such as ribosome stalling via charge interactions of the KKYK motif with the ribosome exit tunnel, which is a phenomenon unrelated to bypassing. However, once these interactions form, they become essential to hold the peptidyl-tRNA on the ribosome during sliding, in line with their crucial role in bypassing.

Our data, in conjunction with previous reports, suggest the following model of programmed ribosome bypassing (Figure 6). Ribosomes translating ORF1 start to slow down at about codon 20, which is regulated by the nascent peptide interaction with the ribosome tunnel. This is important to prevent ribosome pile-up after the leading ribosome pausing at the take-off codon, which would prevent formation of secondary structure elements behind the ribosome. Upon arrival at the take-off codon, interactions of the nascent peptide with the exit tunnel induce the catalytically inactive conformation of the peptidyl transferase center that inhibits translation termination of stop-codon readthrough (21) and together with A-site SL induce formation of an unusual hyper-rotated ribosome conformation (13,22), preparing the ribosome for take-off. An EF-G-catalyzed pseudo-translocation event, employing an mRNA structure formed in the A site as a tRNA mimic, initiates sliding (21,22). While the ribosome moves along the mRNA, 99% have enough energy to move forward to the next nucleotide (Figure 4 and (14)). However, a 1% drop-off fraction is sufficient to explain why only about half of the ribosomes arrive at the landing site. Sliding is guided by interactions with the 5′SL and take-off SL elements formed behind the ribosome and is terminated with the help of the 3′SL element (13,14). Programmed bypassing is strongly temperature-dependent. At lower temperatures, fewer ribosomes adopt the hyper-rotated ribosome conformation that initiates the take-off (21,22), which disfavors efficient sliding (Figure 5).

In addition to offering mechanistic information on the programmed bypassing, the present data provide the first quantitative estimations that may be relevant for the spontaneous sliding. When the non-coding gap exceeds the naturally selected length of 50 nt, ribosomes lose support from the regulatory elements and traverse the remaining gap independently, limited only by the 3′SL and the landing site. Spontaneous sliding, unsupported by the regulatory mRNA elements, dramatically reduces the probability of ribosomes reaching the landing site and subsequently decreases bypassing efficiency. Indeed, the relationship between bypassing efficiency and gap length exhibits two distinct phases (Figure 4). In the first phase, the processivity of the ribosomes is constant (99%) and independent of the gap lengths <56 nt, likely because mRNA re-folding behind the ribosome prevents backward motion and aids the ribosome in reaching the landing site. The second phase observed at gap lengths >56 nt shows a dramatic drop in bypassing efficiency with each additional nucleotide. In this second phase, the ribosome may have a propensity to slide in both directions, akin to ribosomal sliding on 3′UTRs during re-initiation or spontaneous sliding through the stop codon (1,5–7). These findings lead us to propose that when ribosome traverses a non-coding gap exceeding 56 nucleotides in length, its movement starts to resemble free one-dimensional diffusion along the mRNA. In this scenario, there is an increased likelihood for the ribosome to either stall or drop off before it reaches the landing site. Conversely, for gaps shorter than 50 nucleotides, ribosome sliding does not exhibit characteristics of free diffusion. Instead, it appears to be a restricted process, guided by regulatory elements. These elements are instrumental in ensuring a high efficiency of sliding across the non-coding gap. This distinction between the two scenarios underscores the critical role of gap length and regulatory elements in influencing ribosome processivity and sliding behavior along mRNA.

Spontaneous ribosome sliding through the 3′ UTR is likely to be infrequent and limited to certain mRNA contexts. Our data indicate that in the absence of supporting mRNA structures, the likelihood of ribosome drop-off from the track increases significantly with each additional nucleotide, predicting that sliding would be possible only over short distances. Moreover, these tracks must lack secondary structure elements, because those can cause ribosome slowdown and drop-off (14). In fact, ribosome movement along mammalian 3′UTRs is influenced by mRNA sequence, with higher GC content suppressing post-termination ribosome migration (35). A similar type of movement may be prevalent during re-initiation on bacterial polycistronic genes, especially when the initiation codon of an internal gene overlaps with the termination region of the preceding gene in an operon. In such cases, the ribosome recycling factor may not release ribosomes from the gene junction (4), enabling them to find the new start site by sliding. It remains unclear whether other features of spontaneous sliding in the cell are represented by the observed low-efficiency sliding beyond 56 nt of gap lengths. Because spontaneously sliding 70S ribosomes are likely in a post-termination state, they must be capable of recruiting EF-G, which normally facilitates ribosome splitting with the help of RRF in the subsequent ribosome recycling step. If RRF binding failed, EF-G recruitment and GTP hydrolysis could promote sliding similar to what has been observed on *gene 60*. It is also possible that the tendency to slide could be influenced by the local mRNA structures that spontaneously fold in the A site; these aspects should be addressed in future research. The mRNA context, folding, and potentially the nature of the nascent peptide, as well as environmental conditions may influence spontaneous sliding, as well as the timing and accuracy of ribosome switching to translation. Despite its potential role in regulating, translation, sliding remains poorly understood due to the short lifetimes of sliding and the heterogeneity of sliding ribosomes. This study represents one of the initial attempts to elucidate the principles of ribosome sliding along mRNA.

## Supplementary Material

gkae396_Supplemental_File

## Data Availability

All data needed to evaluate the conclusions in the paper are present in the paper and/or the Supplementary Materials.
