# Supplementary material for: Kinetics of programmed and spontaneous ribosome sliding along the mRNA
Source: Nucleic Acids Res. 2024 May 23;52(11):6507–17. doi: 10.1093/nar/gkae396 (PMC11194080; doi:10.1093/nar/gkae396)
Supplement: gkae396_Supplemental_File [file gkae396_supplemental_file.pdf]

Supplementary Table and Figures for

Kinetics of programmed and spontaneous ribosome sliding along the mRNA

Tamara Senyushkina, Ekaterina Samatova, Maria Klimova, and Marina V. Rodnina*

Max Planck Institute for Multidisciplinary Sciences, Department of Physical Biochemistry,
37077 Göttingen, Germany

Supplementary Table 1. Kinetics of *gene 60* translation in HiFi and $TAKM_7$ buffers

Translation time courses were evaluated by fitting to a delay (t_{delay}) followed by exponential increase in product formation (t_{exp}), which together yield the total synthesis time (t_{total}). The t_{gap} value is calculated from t_{total} (ORF1+ORF2) – t_{total} (Δgap). The average elongation rate is calculated as length of peptide in aa / t_{total}.

mRNA	t_{delay}, s	t_{exp}, s	t_{total}, s	t_{gap}, s	k_{el}, aa/s
ORF1, HiFi	3.2 ± 0.2	3.9 ± 1.5	7.1 ± 0.4		6.3 ± 0.3
ORF1+ORF2, HiFi	44.3 ± 1.5	32.0 ± 2.7	76.3 ± 3.1	44.6 ± 6.2	2.1 ± 0.1
Δgap, HiFi	17.8 ± 3.3	13.9 ± 4.3	31.7 ± 5.4		5.0 ± 0.6
ORF1, $TAKM_7$	6.9 ± 0.3	8.8 ± 2.1	15.7 ± 2.1		2.9 ± 0.4
ORF1+ORF2, $TAKM_7$	63.9 ± 2.3	88.5 ± 8.0	152.4 ± 8.3	99.8 ± 8.5	1.0 ± 0.1
Δgap, $TAKM_7$	38.6 ± 0.6	14.0 ± 1.6	52.6 ± 1.7		3.0 ± 0.1

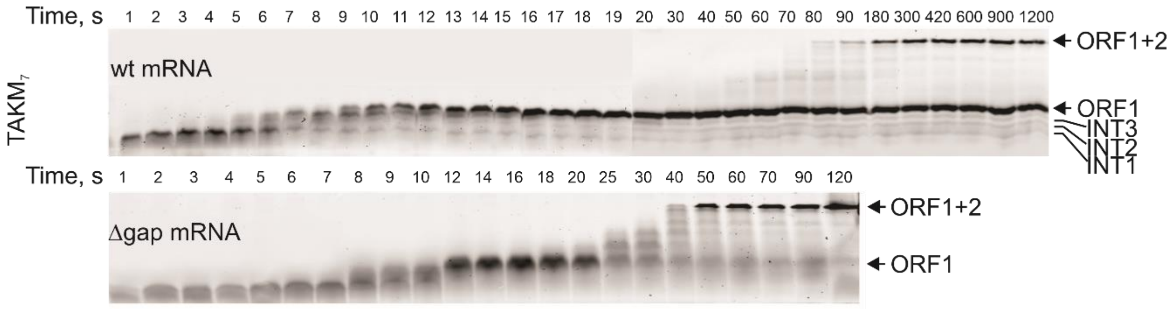

Supplementary Fig. 1. Bypassing in $TAKM_7$.

Time courses of translation for the full-length wt and Δgap mRNAs in $TAKM_7$. Translation products were separated by SDS-PAGE and detected using the fluorescent reporter (BodipyFL) attached to the N-terminal Met in the peptide. Translation of ORF1 is terminated at the UAG stop (46 aa-long ORF1 product); ORF1+2 is the product of bypassing and translation to the end of the ORF2 (160 aa). Δgap is the construct containing ORF1+ORF2 without the proximal stop codon and the non-coding gap.

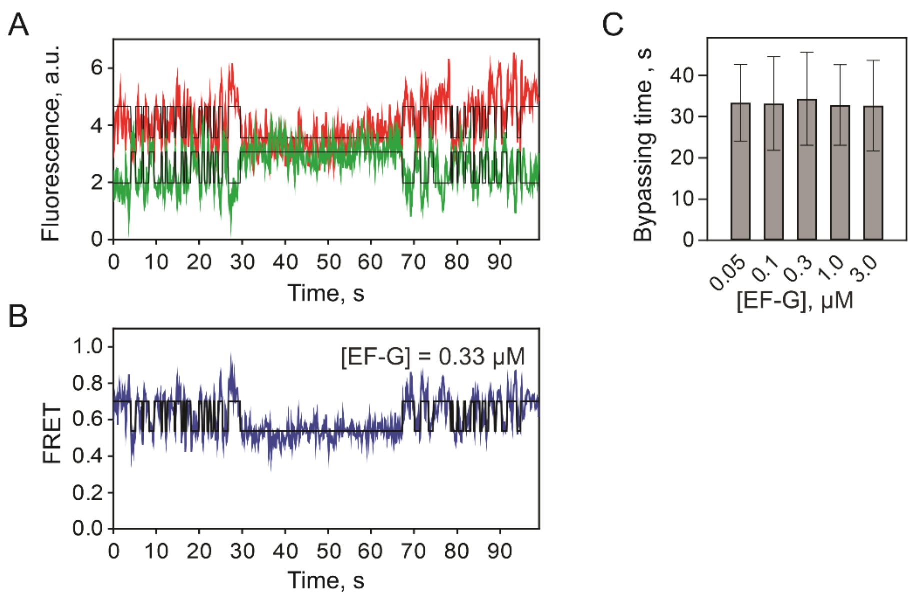

Supplementary Fig 2. Determination of the bypassing time by smFRET.

A. Representative single-molecule fluorescence intensity traces showing donor (green) and acceptor (red) fluctuations between non-rotated and rotated states of the ribosome.

B. smFRET calculated from the time courses shown in A. The trace shows rapid transitions between FRET 0.7 (non-rotated state) and 0.5 (rotated state) representing successive elongation cycles. The long-lived FRET 0.5 state corresponds to bypassing, which includes take-off, sliding and landing. After bypassing, the ribosome resumes rapid translation, as indicated by rapid fluctuations. Black solid line shows the HMM fit of the data.

C. Bypassing time at different EF-G concentrations shown as mean value with error bars representing standard deviation from the following number of traces: N = 61 at [EF-G] = 0.05 µM, N = 181 at [EF-G] = 0.1 µM, N = 305 at [EF-G] = 0.3 µM), N = 241 at [EF-G] = 1.0 µM, and N = 48 at [EF-G] = 3 µM.

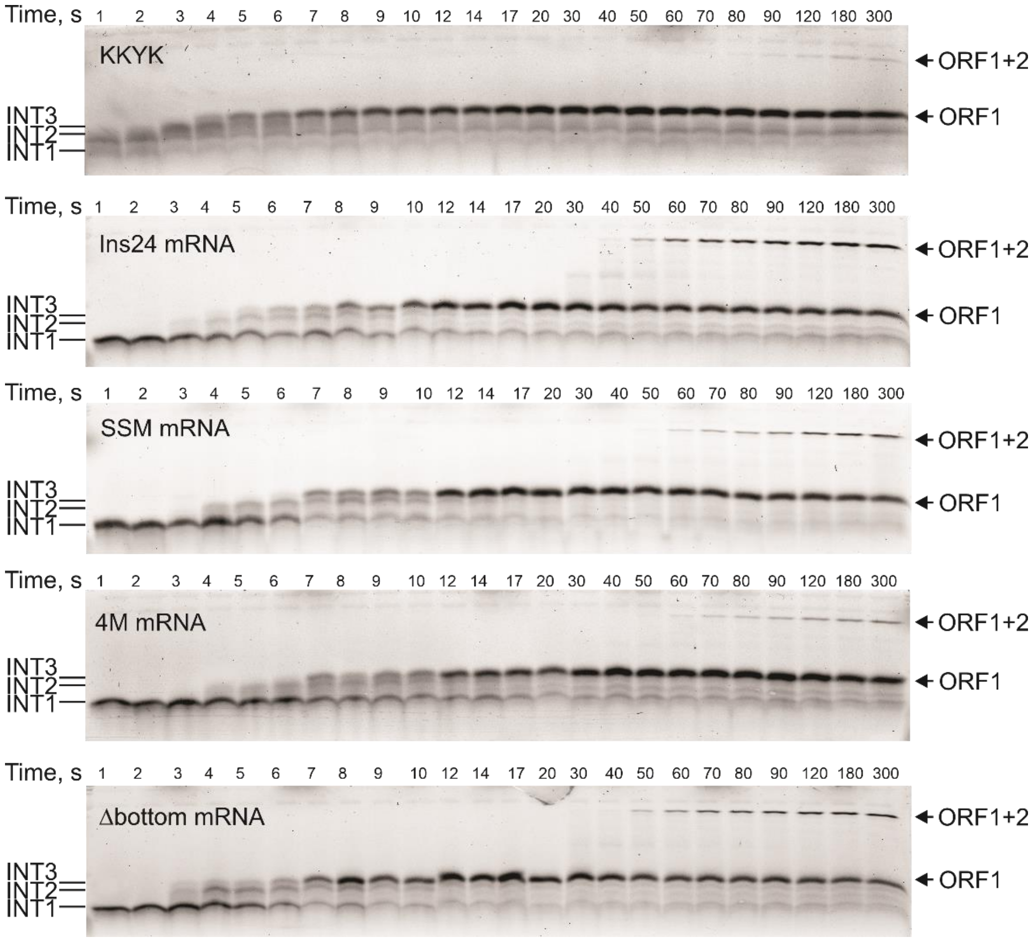

Supplementary Fig. 3. Time courses of ORF1 and ORF1+2 synthesis on mutant mRNA constructs shown in Fig. 3A.
